# Interactive Web-Based Resource for Annotation of Genetic Variants Causing Hereditary Angioedema (HADA): Database Development, Implementation, and Validation

**DOI:** 10.2196/19040

**Published:** 2020-10-09

**Authors:** Alejandro Mendoza-Alvarez, Adrián Muñoz-Barrera, Luis Alberto Rubio-Rodríguez, Itahisa Marcelino-Rodriguez, Almudena Corrales, Antonio Iñigo-Campos, Ariel Callero, Eva Perez-Rodriguez, Jose Carlos Garcia-Robaina, Rafaela González-Montelongo, Jose Miguel Lorenzo-Salazar, Carlos Flores

**Affiliations:** 1 Research Unit Hospital Universitario Nuestra Señora de Candelaria Universidad de La Laguna Santa Cruz de Tenerife Spain; 2 Genomics Division Instituto Tecnológico y de Energías Renovables Santa Cruz de Tenerife Spain; 3 CIBER de Enfermedades Respiratorias Instituto de Salud Carlos III Madrid Spain; 4 Allergy Unit Hospital Universitario Nuestra Señora de Candelaria Universidad de La Laguna Santa Cruz de Tenerife Spain; 5 Instituto de Tecnologías Biomédicas Universidad de La Laguna Santa Cruz de Tenerife Spain

**Keywords:** genetic cause, hereditary angioedema, knowledge database, precision medicine, variant interpretation

## Abstract

**Background:**

Hereditary angioedema is a rare genetic condition caused by C1 esterase inhibitor deficiency, dysfunction, or kinin cascade dysregulation, leading to an increased bradykinin plasma concentration. Hereditary angioedema is a poorly recognized clinical entity and is very often misdiagnosed as a histaminergic angioedema. Despite its genetic nature, first-line genetic screening is not integrated in routine diagnosis. Consequently, a delay in the diagnosis, and inaccurate or incomplete diagnosis and treatment of hereditary angioedema are common.

**Objective:**

In agreement with recent recommendations from the International Consensus on the Use of Genetics in the Management of Hereditary Angioedema, to facilitate the clinical diagnosis and adapt it to the paradigm of precision medicine and next-generation sequencing–based genetic tests, we aimed to develop a genetic annotation tool, termed Hereditary Angioedema Database Annotation (HADA).

**Methods:**

HADA is built on top of a database of known variants affecting function, including precomputed pathogenic assessment of each variant and a ranked classification according to the current guidelines from the American College of Medical Genetics and Genomics.

**Results:**

HADA is provided as a freely accessible, user-friendly web-based interface with versatility for the entry of genetic information. The underlying database can also be incorporated into automated command-line stand-alone annotation tools.

**Conclusions:**

HADA can achieve the rapid detection of variants affecting function for different hereditary angioedema types, and further integrates useful information to reduce the diagnosis odyssey and improve its delay.

## Introduction

Hereditary angioedema (HAE) is a rare genetic disease caused by an increase of vascular permeability, generating recurrent acute swelling episodes commonly localized on the face, trunk, and extremities. HAE can also be life-threatening when the upper airways or the tissues from the oral cavity are affected [1,2]. Because of its nonspecific clinical signs, HAE is poorly recognized by physicians. Therefore, delayed or ambiguous diagnoses are common, increasing the risk of patient morbidity and mortality [3]. With exceptions for particular subtypes, the recent international consensus guidelines recommend the use of genetic testing to reach a definitive HAE diagnosis in clinical practice [4]. However, with increasing recognition that the genetic cause of HAE is more complex than previously anticipated [5], the tasks involved in the identification of the genetic defect in each patient are increasingly demanding [6].

With the decreasing cost and increasing sequencing throughput of next-generation sequencing (NGS), the screens for gene defects can now be accomplished through a simultaneous evaluation of gene sets, the exome, or the whole genome as indicated [7]. However, access to either high-performance computational equipment and trained bioinformatics personnel or, alternatively, to paywalled cloud-based all-inclusive solutions is the most limiting factor for NGS to be efficiently used in clinical settings [5]. To facilitate the identification of pathogenic variants in HAE patients, Kalmár et al [8] developed HAEdb, an online locus-specific database to centralize the information of the genetic alterations, which allows researchers to retrieve mutation information and contribute new detected variants. However, HAEdb focuses only on the most frequently affected gene (*SERPING1*, encoding the C1 esterase inhibitor [C1-INH]) and uses a data retrieval scheme based on matching with the existing records, requiring the user to prioritize the most likely variant affecting function. Besides, HAEdb has not been updated to include the pathogenic classification according to the standard guidelines established by the American College of Medical Genetics and Genomics (ACMG) [9], which would allow for standardization between laboratories and studies, while guiding global efforts for improving NGS-based diagnosis to move toward the precision medicine paradigm. More recent efforts such as that of Ponard et al [10] have aimed to fill this gap and updated the mutational spectrum in two of the known HAE genes (*SERPING1* and *F12*) based on the Leiden Open Variant Database (LOVD) v3.0, a platform-independent framework for the maintenance and curation of a web-based database of genetic variants [11]. Nevertheless, these resources are ill-adapted to current NGS technologies and to the evolving knowledge of the genetic causes of HAE.

To fill this gap, we here present the Hereditary Angioedema Database Annotation (HADA) tool, a freely accessible, user-friendly, and versatile web-based interface to facilitate the identification of the genetic variants causing HAE.

## Methods

### Gene and Variant Extraction to Build the Database

**Figure 1:**
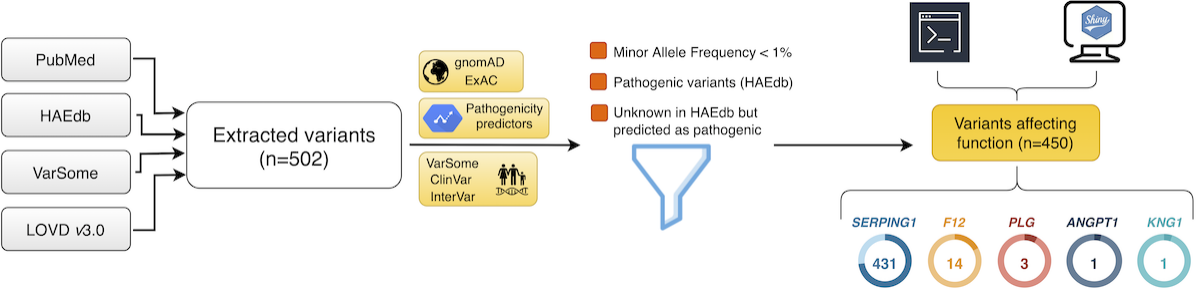
Schematic representation of the steps involved in gene and variant extraction, annotation, and database curation. All genetic variants reported in the articles studying hereditary angioedema (HAE) families in PubMed, HAEdb, VarSome, and the Leiden Open Variation Database (LOVD) were collected (n=502). ANNOVAR was used for annotation of frequencies, pathogenic predictors, and pathogenic classifications, among other information. Variants with a minor allele frequency below 1%, declared as pathogenic in HAEdb or as unknown but with a pathogenic prediction were kept as the set of known variants affecting function (n=450).

We retrieved the variants found among HAE cases and relatives from HAEdb [8] (accessed June 18, 2020) and LOVD v3.09 (Figure 1). These databases contain hundreds of records for variants in the *SERPING1* or *F12* genes detected in clinical studies of families with one or more members affected by HAE. Additionally, a search was performed on January 1, 2020 in PubMed for the terms “angioedema” and “mutation” with the aim of retrieving other HAE genes from the literature. From this search, studies focusing on acquired forms of angioedema were ruled out, and an in-depth analysis of each prioritized study was performed. The VarSome database [12] was also screened to retrieve variants, as well as to update the genetic nomenclature of variant descriptors. The information described in the corresponding articles where each variant was described was manually inspected to verify that the original descriptions were accurate.

Given that a confident clinical interpretation of structural variants is challenging and that the standards for reporting those involving copy number variants have only recently been set [13], the criteria indicated above were only applied to single nucleotide variants (SNVs) and small insertion/deletions (indels). Therefore, the 45 gross mutations (ie, structural variants in the *SERPING1* gene) from HAEdb that have been identified in HAE families were not integrated into HADA.

### Database Variant Annotation

According to their chromosomal coordinates, the selected variants were first adapted to the GRCh37/hg19 reference genome, exonic locations, Human Genome Variation Society (HGVS) nomenclature, coding effect, and PubMed citation. ANNOVAR v18.04.16 [14] was used to annotate according to RefGene, the allele frequency in gnomAD v2.1.1 [15] and ExAC [16] (November 29, 2015 release), dbSNP build 150 information, and precalculated pathogenicity predictors (SIFT, PolyPhen2, MutationTaster, CADD, DANN, MetaSVM, LRT, and phastCons mammalian). Pathogenic probabilities according to ClinPred [17] and the ACMG pathogenic classification as determined by ClinVar [18] (March 5, 2019 release), InterVar [19] (January 18, release), and VarSome (accessed June 13, 2020) were also annotated (Figure 1).

### Database Curation

Many original descriptions of the genetic studies in HAE did not clearly declare the causality of the reported variants, thereby increasing the difficulty to identify variants with effects on the disease as opposed to variants without a disease effect [4]. To facilitate the interpretation of HADA results, we imposed the following filters for a variant to be designated as a variant affecting function (Figure 1): (1) variants described as pathogenic in HAEdb; (2) variants with unknown effects in HAEdb but predicted as pathogenic, likely pathogenic, or of uncertain significance (VUS) by VarSome, ClinVar, or InterVar (a flag will advise users in the case that contradictory classification information is reported for a variant); and (3) a reported minor allele frequency<1% in gnomAD, either on the overall sample or for non-Finish Europeans (because most of the genetic studies in HAE to date have focused on European families).

Finally, to facilitate the identification of potentially novel variants in the user’s uploaded data, HADA also includes all SNVs and small indels that were not classified as a variant affecting function using the criteria indicated above from dbSNP build 150, ClinVar, and InterVar located within 50-bp flanking regions of all exons from known HAE genes.

### Implementation of HADA

**Figure 2:**
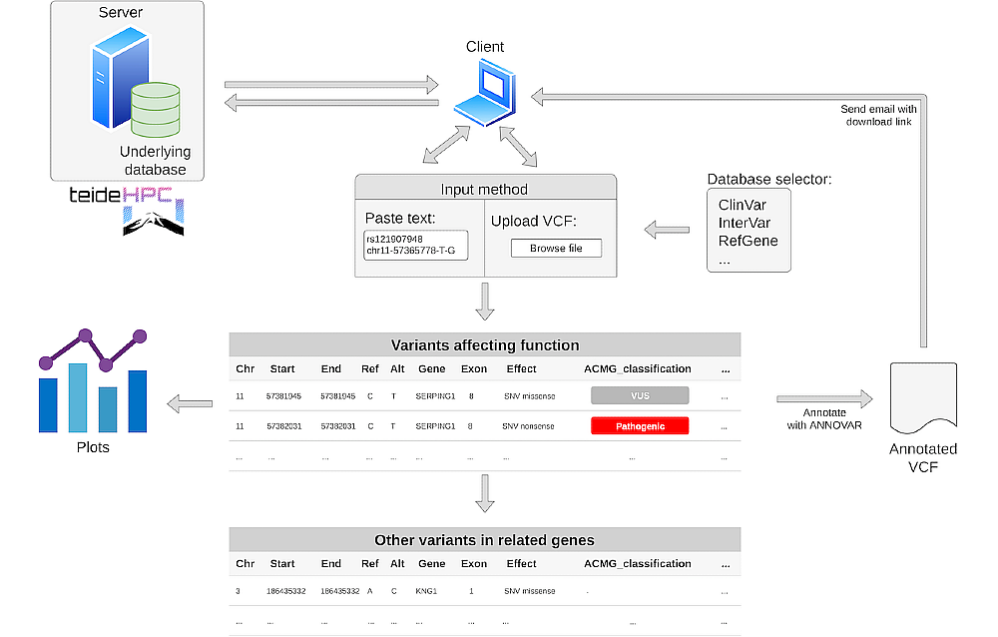
Schematic representation of the HADA architecture and user interface. HADA is hosted on a server at TeideHPC, through a UI-based on Shiny. When the user sends a query to the server, it is encrypted and interrogates the HADA database, and returns matched variants affecting function associated with hereditary angioedema. The app also searches the data for other variants located within known hereditary angioedema genes to facilitate the identification of potentially novel variants. Once finished, an email with the link to download the annotated variant calling file (VCF) is sent to the user. At the end of the process, uploaded and annotated files are deleted from the server.

The database is built on MongoDB v4.2.1, a NoSQL query engine, to speed up database user queries and the variant calling file (VCF)-oriented analysis (Figure 2). HADA is built in Shiny v1.3.2, an R v3.6.1 package (R Foundation for Statistical Computing, Vienna, Austria) for building web apps. Specifically, we used ShinyJS v1.0 to run JavaScript code within the web app frontend and Plotly v4.9.0 to generate interactive plots. ANNOVAR v18.04.16 is used to provide annotations from the database in the uploads. To preserve potentially sensitive information included in the uploaded VCFs, HADA uses an encrypting code based on Cryfa [20] that automatically secures the access to the file and decrypts it once returned to the user. This provides a high level of security and transfers the data control to the user. No sensitive sample information is stored or maintained in the server.

The curated database used by HADA is also available as a separate download at github [21] and it has been configured to annotate VCFs using ANNOVAR from the command line interface tool so that it can be easily incorporated into routine standalone NGS bioinformatics workflows. The HADA web interface is publicly accessible [22] hosted on TeideHPC premises [23].

### Validation in an HAE Family

To validate HADA with comprehensive NGS data, we generated whole-exome sequencing (WES) data from a patient with HAE and his two family members recruited by the Allergy Service from the Hospital Universitario Nuestra Señora de Candelaria (HUNSC), Santa Cruz de Tenerife, Spain. The study was approved by the HUNSC Ethics Committee and written informed consent was obtained from the patients. The index patient was a 31-year-old male who visited the emergency department more than 5 times due to acute angioedema attacks with facial and cutaneous symptoms, manifesting as episodes affecting the upper airways from the age of 23 years. His biochemical blood test showed normal levels of C1-INH (47 mg/dL) based on the N Antisera to Human Coagulation Factors and C1 Inhibitor kit (Siemens Healthcare Diagnostics, Marburg) and reduced C1-INH activity (10%) (Berichrom C1 inhibitor, Siemens Healthcare Diagnostics, Marburg, Germany), suggesting HAE type II. His mother (60 years old) and a sister (23 years old) reported no symptoms of HAE, and also consented to participate in the study.

Briefly, sequencing libraries were prepared from DNA extracted using a commercial column-based kit (GFX kit, GE Healthcare, Little Chalfont, UK) using Nextera DNA Exome Kit (Illumina Inc, San Francisco, CA). The TapeStation 4200 system (Agilent Technologies, Santa Clara, CA) was used for library size estimation and the concentration was determined by the Qubit dsDNA HS Assay (Thermo Fisher Scientific, Waltham, MA). Libraries were sequenced on a HiSeq 4000 Sequencing System (Illumina Inc) with paired-end 75-base reads. Libraries were sequenced along with 1% of PhiX control V3 to an average depth of 50X after removal of duplicate reads. Sequencing reads were preprocessed with bcl2fastq v2.18 and mapped to hg19/GRCh37 with the Burrows-Wheeler Aligner 0.7.15-r1140 [24]. Resulting BAM files were processed with Qualimap v2.2.1 [25], SAMtools v1.3 [26], and Picard v2.10.10 [27]. Variant calling was performed using HaplotypeCaller following the Best Practices workflow recommendations for germline variant calling in GATK (v3.8) [28], obtaining one VCF for each sequenced individual. A high confidence set of variants was obtained after applying the following filters: variants with PASS, read depth≥20, genotype quality≥100, mapping quality≥50. The transition-to-transversion nucleotide substitution ratio was also obtained as a quality control for the refined set of variants. Sequencing data and variant identification were obtained by the Instituto Tecnológico y de Energías Renovables (Santa Cruz de Tenerife, Spain).

## Results

### Overall Characteristics of the Database

**Figure 3:**
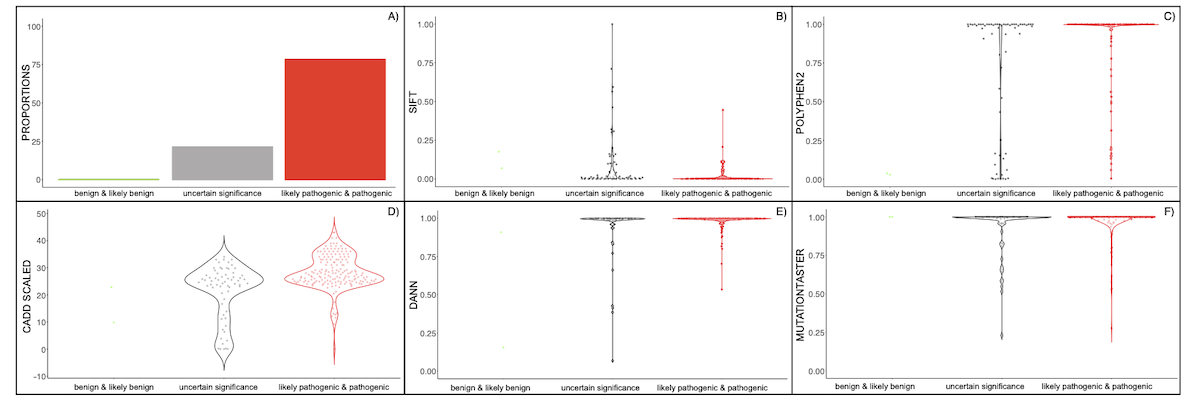
Precalculated pathogenic scores by the American College of Medical Genetics and Genomics (ACMG) pathogenic class for variants affecting function recorded in HADA. Panel A: VarSome proportions of ACMG classes among the hereditary angioedema variants affecting function. Panels B–F: pathogenicity prediction scores by ACMG pathogenic classes as provided by VarSome.

A total of 502 variants from 5 genes causally linked to HAE were retrieved from PubMed, HAEdb, VarSome, and LOVD. A subset of 450 variants was designated as variants affecting function, including variants reported in the following 5 genes: *SERPING1* (n=431), *F12* (n=14), *PLG* (n=3), *ANGPT1* (n=1), and *KNG1* (n=1) (Figure 1). Three of these genes were discovered in the last 2 years based on WES approaches in HAE patients without C1-INH defects but not carrying *F12* variants affecting function [29-31]. ClinVar offered very limited information on this set of variants affecting function, as only 34 of them (7.6%) had corresponding ACMG class assignment: 2 are reported as benign or likely benign, 6 are indicated as VUS, and 26 are classified as pathogenic and likely pathogenic. InterVar included information for half of the set (226/450, 50.2%). However, 171 (75.7%) of these were classified as VUS. VarSome was the only resource that allowed assigning ACMG classes to all retrieved variants affecting function. According to VarSome, most of the HAE variants affecting function are classified as pathogenic (183/450, 40.6%) or likely pathogenic (171/450, 38.0%) (Figure 3). Although VarSome did not classify any of the HAE variants affecting function as benign or likely benign, 96 of them (21.3%) were still reported as VUS. Besides, precalculated pathogenicity predictors were available for a mean of 243 of the variants affecting function in the database. Taken together, these results highlight the existing gap in current interpretations of variant pathogenicity [32,33].

### HADA Interface and Usage

**Figure 4:**
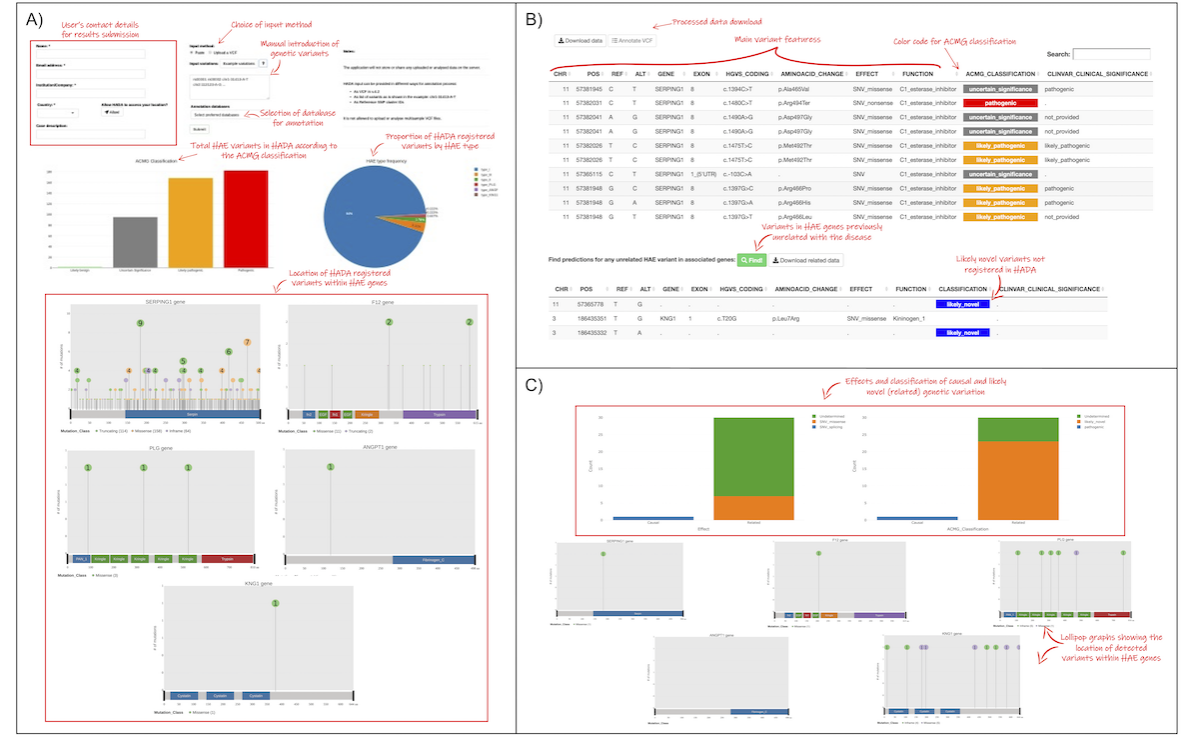
Selected snapshots of the HADA graphical interface with step-by-step instructions. Users can query variants of interest or alternatively upload an individual variant calling file (VCF) in the home page (A). The detected variants tab (B) shows the existence of variants affecting function in the query, as well as other variants in hereditary angioedema (HAE) genes. Plots are also generated and available for users to download (C).

HADA has been developed as a user-friendly graphical web-interface tool. It is designed with the objective of enabling the analysis of genetic variants regardless of the detection approach used for the screening of the genetic causes of HAE (Figure 4). As such, HADA is compatible with individual VCFs that have been obtained at any scale (gene panel, exome, or whole genome) and by any NGS technology. Alternatively, the user could opt to provide hg19 chromosome coordinates of the variant(s) of interest as the rsID number, HGVS coordinate, or amino acid change, among others. The latter is still a common standard in laboratories relying on Sanger sequencing results. All of the annotation information used to construct the underlying database can be selected by the user to extract the available information. To facilitate a prospective analysis of each query variant, references to the corresponding articles reporting the variant are also offered (in chronological order). Direct links to external tools (ie, VarSome), which allows accessing graphical information of gene transcripts, complement the possibilities of HADA. Despite the fact that sensitive patient information is not necessary for HADA to provide results, HADA integrates an automatic data encryption algorithm for the queries based on VCF data, ensuring a password-secured encryption of the input data while uploaded and of the decryption when results are downloaded. Furthermore, the information provided by the user is not stored permanently by the server.

The data processing workflow is shown in Multimedia Appendix 1. Once the data are uploaded, the variants contained in the VCF or that were provided by the user in chromosomal coordinates are matched against HADA. Automated detection and information extraction occur on the fly. Matched variants are subsequently shown in tabular format, accompanied by information previously selected by the user. HADA provides the ACMG classifications for each variant according to a color key to facilitate the identification of the HAE variants affecting function in the queries. HADA also suggests the subtype of HAE for which the variant affecting function is involved.

We anticipate that a user query might involve variants that have not yet been described among HAE genes (ie, novel HAE-related variants) or that have not been described in the scientific literature as an affecting functional variant to date. HADA integrates a genomic coordinates file that allows for the retrieval of predictions for any variant with any reference/alternative allele combination in any exonic region (plus a 50-bp flanking region on both ends of each locus) of the known HAE genes. Query results provided by HADA can be downloaded by the user in plain-text format in a comma-separated file, or as an annotated VCF in cases in which a VCF was uploaded by the user.

Finally, publication-ready plot generation and downloading are also incorporated in HADA. These functions plot graphs that summarize the information obtained from input data (ie, a VCF), as well as charts summarizing the proportions of the ACMG pathogenic classes predicted by VarSome, ClinVar, and InterVar separately, and the subtype of HAE that is associated with a variant affecting function that is identified in the query.

### Validation of HADA in an HAE-Affected Family

To demonstrate the utility of HADA for the NGS-based identification of the variant affecting function involved in an HAE type II family, WES data (45 Mb) from the index patient, and the unaffected mother and sister were obtained. This detected a mean of 16,650 high-confidence variants per individual. Sequencing metrics and variant calling results are shown in Table 1. Resulting individual VCFs were processed with HADA, which reported a variant c.1396C>A (rs28940870) within *SERPING1* both in the index case and the sister that was heterozygous in both cases (compatible with an autosomal dominant inheritance pattern). The depth of coverage at this locus was 72X and 69X, respectively.

**Table 1:** Summary of the sequencing results in the validation study.

Feature		Index	Sister	Mother
Median insert size (bp^a^)		297	268	268
Total reads (millions)		130.4	100.5	148.7
Aligned reads (%)		99.5	100	100
Mean coverage (%)		49.3	44.3	54.2
Targeted fraction ≥ 30X (%)		66	55	76
Ti/Tv^b^		3.16	3.19	3.22
**Number of variants^c^**		16,394	16,231	17,325
	SNVs^d^	16,087	15,917	16,984
	Indels^e^	307	314	341

^a^bp: base pairs.

^b^Ti/Tv: ratio of transitions to transversions.

^c^High confidence variants (FILTER=PASS; total depth≥20; genotype quality≥100; and mapping quality ≥ 50).

^d^SNVs: single nucleotide variants.

^e^Indels: insertion/deletion.

Most pathogenic prediction scores provided by HADA supported the variant as deleterious/damaging, and ACMG class predictors classified it either as a pathogenic/likely pathogenic variant or VUS (Table 2). This variant predicts an amino acid change (p.Arg466Ser) affecting the catalytic center of the protein, which has been previously described in independent HAE type II cases and was shown to be responsible for reduced C1-INH activity [34-36]. These observations are fully compatible with the clinical and biochemical findings supporting a diagnosis of HAE type II in the index case. Surprisingly, the sister was asymptomatic and had never experienced angioedema attacks, which further exemplifies the incomplete penetrance of HAE or that disease onset has not yet occurred. In any case, the provided genetic information could be used to anticipate potential clinical symptoms.

**Table 2:** American College of Medical Genetics and Genomics (ACMG) class predictions and pathogenic scores provided by HADA for the c.1396C>A variant found in a hereditary angioedema–affected family.

Predictor		Estimation
**ACMG class prediction**		
	VarSome	VUS^a^
	ClinVar	Pathogenic
	InterVar	Likely pathogenic
**Pathogenic predictors**		
	SIFT^b^	Damaging
	Polyphen2^c^	Benign
	MutationTaster	Disease-causing
	CADD^d^	31
	DANN^e^ score	0.997
	DANN rankscore	0.798
	LRT^f^	Deleterious
	MetaSVM	Damaging

^a^VUS: variant of uncertain significance.

^b^SIFT: Sorting Intolerant From Tolerant.

^c^PolyPhen2: Polymorphism Phenotyping v2.

^d^CADD: Combined Annotation Dependent Depletion.

^e^DANN: Deleterious Annotation of genetic variants using Neural Networks.

^f^LRT: likelihood ratio test.

^g^MetaSVM: meta-analytic support vector machine.

## Discussion

### Principal Findings

Here, we present HADA, a web-based analysis tool to facilitate the identification of the genetic variants causing HAE. With HADA, we aimed to provide a user-friendly tool to assist in the diagnosis of HAE that is adapted to NGS technologies and to the evolving knowledge of the causes of HAE. HADA has potential to reduce the time to interpret the detected variants in the NGS era by aggregating data from multiple sources, a process that commonly takes several hours to complete if it is handled manually [37,38]. At the moment, HADA integrates information from 450 SNVs and indels from 5 genes (*SERPING1*, *F12*, *PLG*, *ANGPT1*, and *KNG1*) that we classified as variants likely affecting function. While curating this information, we found that variant descriptions in HAE cases did not follow a standard [4], and many of the studies did not clearly declare the causality of the reported variants [35,39]. This situation helps to explain why up to 21.3% (n=96) of the simple bona fide variants affecting function continue to be reported as VUS in the best case. Finally, we demonstrated the utility of HADA for explaining HAE type II in a family that was assessed by WES in three family members, and offered conclusive information about the existence of the previously described variant c.1396C>A (p.Arg466Ser) in *SERPING1*.

As is the case for many other rare diseases, genetic testing in HAE has become an important step to reduce the diagnostic odyssey [40], increase the diagnostic yield, and tailor treatments [4,5]. The widespread adoption of NGS technology in clinical settings has led to the emergence of a wide variety of bioinformatics tools to assist and accelerate the detection and interpretation of associated genetic variants and their impact on disease risks. In recent years, an array of variant prioritizers and interpreters have been developed to obtain optimal rankings for the variants causing the disease. However, NGS-based solutions have not been adopted in the HAE community until recently [29-31]. Part of the explanation may reside in the fact that only two causal genes were known until 2018, and therefore most clinical diagnoses could only be made based on clinical symptoms and the biochemical measurements of C4 and C1-INH levels in plasma, along with C1-INH activity [5]. Importantly, public resource updates to assist in variant interpretation lag behind the pace of genetic discoveries. This is the case of Simply-ClinVar [41] or ClinVar, which are still outdated with respect to the genetic causes of HAE, reporting information for only two of the associated genes (*SERPING1* and *F12*). Simply-ClinVar also suggests *SLC34A1* as another HAE gene, although this evidence is unsupported by the current literature. This situation is changing currently, and the benefits of using NGS technologies to assess multiple genes simultaneously for HAE diagnosis are now clearer [5]. In fact, in patients with normal levels and activity of C1-INH, genetic testing is recommended for the routine diagnosis of many HAE subtypes. Furthermore, according to Germenis et al [4] and the international consensus on genetic aspects of HAE, it is widely recognized that C4 and C1-INH plasma measurements can generate inconclusive results even in patients with genetic defects in C1-INH. Based on this, HADA will surely help practitioners to adapt to the NGS-based genetic screens of HAE cases.

HADA has some strengths and limitations for the interpretation of genetic variants involved in the causes of HAE. Among the limitations, the tool does not currently assess VCFs from families or trios and does not allow inferring whether a variant affecting function has a de novo origin from the patient’s data. Similarly, HADA uses GRCh37/hg19 coordinates, which is still considered to be the gold standard in clinical settings [42]. Despite the fact that HADA can report the existence of variants residing in introns near the key elements for splicing, as has been recently found for a type I HAE case [43,44], novel variants affecting function residing deep within intron positions will remain undetected. The main reason is the limited capacity of current algorithms to predict the pathogenic potential of deep intronic variants [45]. Similarly, a variable proportion of information remains unavailable for many of the variants affecting function collected in the underlying database. For example, allelic frequencies are currently available from gnomAD and ExAC for as few as 11 to 20 (2%-4%) of the variants affecting function in HAE. As an example, the variant detected in the family analyzed in this study is a bona-fide causal variant of HAE. However, there are no records of this variant in the gnomAD or ExAC databases. Similarly, not all variants affecting function in HAE are described in current ClinVar or InterVar versions. Collectively, this situation adds to the challenge of better predicting disease effects of many of the variants that are declared as VUS.

Structural variants have an important role in HAE due to the presence of Alu element–related reorganizations affecting function in *SERPING1*, potentially causing HAE type I [46,47]. However, structural variants will not be interpreted by HADA at the moment as they have not been collected in the database. One of the main reasons for this is that, unlike the case for SNVs and small indels, current locus-oriented databases such as HAEdb do not provide the chromosomal coordinates of recorded structural variants from HAE families, making it difficult to integrate in HADA.

Among the strengths of HADA, we highlight the versatility of the tool to allow performing simple queries or to directly upload VCFs. This offers the possibility of analyzing a variant detected at any throughput scale, using either NGS technologies or Sanger sequencing. This feature is a great advantage compared to HAEdb or LOVD, which are not adapted for the automated analysis of NGS data (eg, from a VCF), serving only for single-variant queries, and neither of these previous tools has been subjected to a curation process or the inclusion of novel HAE genes. In addition to the imposed filters to construct the curated database of HADA, allele frequencies are also provided for all populations that are currently collected in the gnomAD and ExAC population databases. To cover the possibility of new discoveries, HADA also includes information from all other described variants in the coding regions, the 5′ and 3′ untranslated regions, and the 50-bp flanking regions (where most splicing variants affecting function are located) to easily flag novel variants in the uploaded data. Finally, despite the fact that its use does not require sensitive information for the analysis, HADA automatically conducts a password-secured encryption of the data and does not permanently store any data on the server.

We anticipate that HADA will be constantly updated to register new variants affecting function and associated information, changes in the pathogenic classification, and the possibility to assess structural variants, and GRCh38/hg38 coordinate and reference sequence number conversions. This will be based on third-party requests and internal updates according to novel discoveries and standards.

### Conclusions

To adapt the genetic diagnosis of HAE to the era of NGS-based genomic medicine, we have developed HADA as a free and publicly available tool for simplifying the identification of simple variants affecting function in HAE. HADA will allow users to focus on biologically relevant questions instead of having to learn to install software dependencies, variant annotation tools, and become familiar with the UNIX command line. The main advantages of HADA are that it is focused on a disease, its ease of use, the ability to display specific and curated information of HAE either from individual or VCF queries, and that it is freely available. By combining these features into a single graphical and interactive tool, we expect that variant prioritization in HAE will become easier, faster, and standardized.

## Appendix

Multimedia Appendix 1 Schematic representation of the variant calling file (VCF) variant matching against the curated database and data downloading in HADA.

## Abbreviations

ACMG: American College of Medical Genetics and Genomics
C1-INH: C1 esterase inhibitor
HADA: Hereditary Angioedema Database Annotation
HAE: hereditary angioedema
HGVS: Human Genome Variation Society
HUNSC: Hospital Universitario Nuestra Señora de Candelaria
indel: insertion/deletion
LOVD: Leiden Open Variant Database
NGS: next-generation sequencing
SNV: single nucleotide variant
VCF: variant calling file
VUS: variant of uncertain significance
WES: whole-exome sequencing
