# Supplementary figures and images for: Interactive Web-Based Resource for Annotation of Genetic Variants Causing Hereditary Angioedema (HADA): Database Development, Implementation, and Validation
Source: J Med Internet Res. 2020 Oct 9;22(10):e19040. doi: 10.2196/19040 (PMC7584987; doi:10.2196/19040)

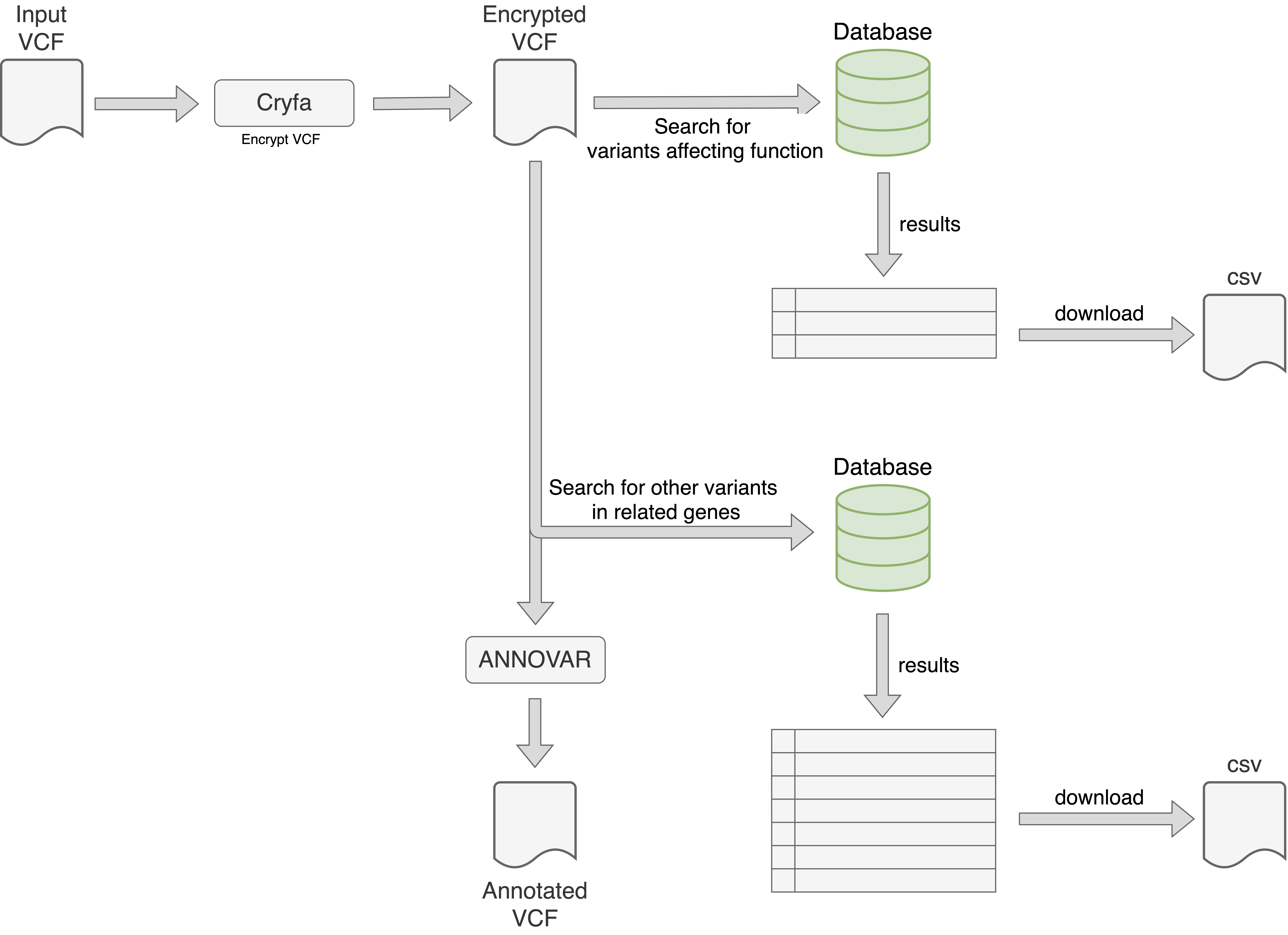

Supplement: Multimedia Appendix 1 [file jmir_v22i10e19040_app1.png]
